# Intestinal pseudo‐obstruction: Unusual presentation of systemic lupus erythematous

**DOI:** 10.1002/ccr3.3907

**Published:** 2021-02-08

**Authors:** Myriam Ayari, Abdelwaheb Nakhli, Zeineb Teyeb, Imen Abdelaali, Syrine Bellakhal, Taieb Jomni

**Affiliations:** ^1^ Gastroenterology Unit Internal Medicine Department La Marsa Internal Security Forces Hospital Tunis Tunisia

**Keywords:** dysmotility, intestinal pseudo‐obstruction, lupus, systemic disease

## Abstract

Systemic diseases should be always considered when managing unexplained intestinal pseudo‐obstruction. Intestinal pseudo‐obstruction related to systemic lupus erythematosus is often responsive to corticosteroid therapy when promptly treated.

## Graphical abstract

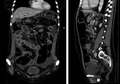

## 1 INTRODUCTION

Intestinal pseudo‐obstruction (IPO) is defined as an intestinal obstruction without mechanical obstructive lesion. It is a rare complication of systemic lupus erythematosus (SLE). We report a case of SLE inaugurated by IPO to emphasize the importance of early recognition of the diagnosis especially that SLE‐related IPO responds well to corticosteroid therapy.

Intestinal pseudo‐obstruction (IPO) is a gut motility disorder resulting from intestinal peristalsis impairment leading to severe obstructive symptoms without mechanical causes. Rarely, it can be a manifestation of systemic lupus erythematosus (SLE).^1^ Prevalence of IPO in patients with SLE is about 2%.^2^ SLE‐related IPO is associated with increased morbidity and mortality, mostly if misdiagnosed and incorrectly treated.^2^ Here, we report a case of IPO as first manifestation of SLE to highlight the importance of early recognition of the diagnosis and so avoid unnecessary surgery especially that SLE‐related IPO responds well to corticosteroids.

## 2 CASE PRESENTATION

**FIGURE 1:**
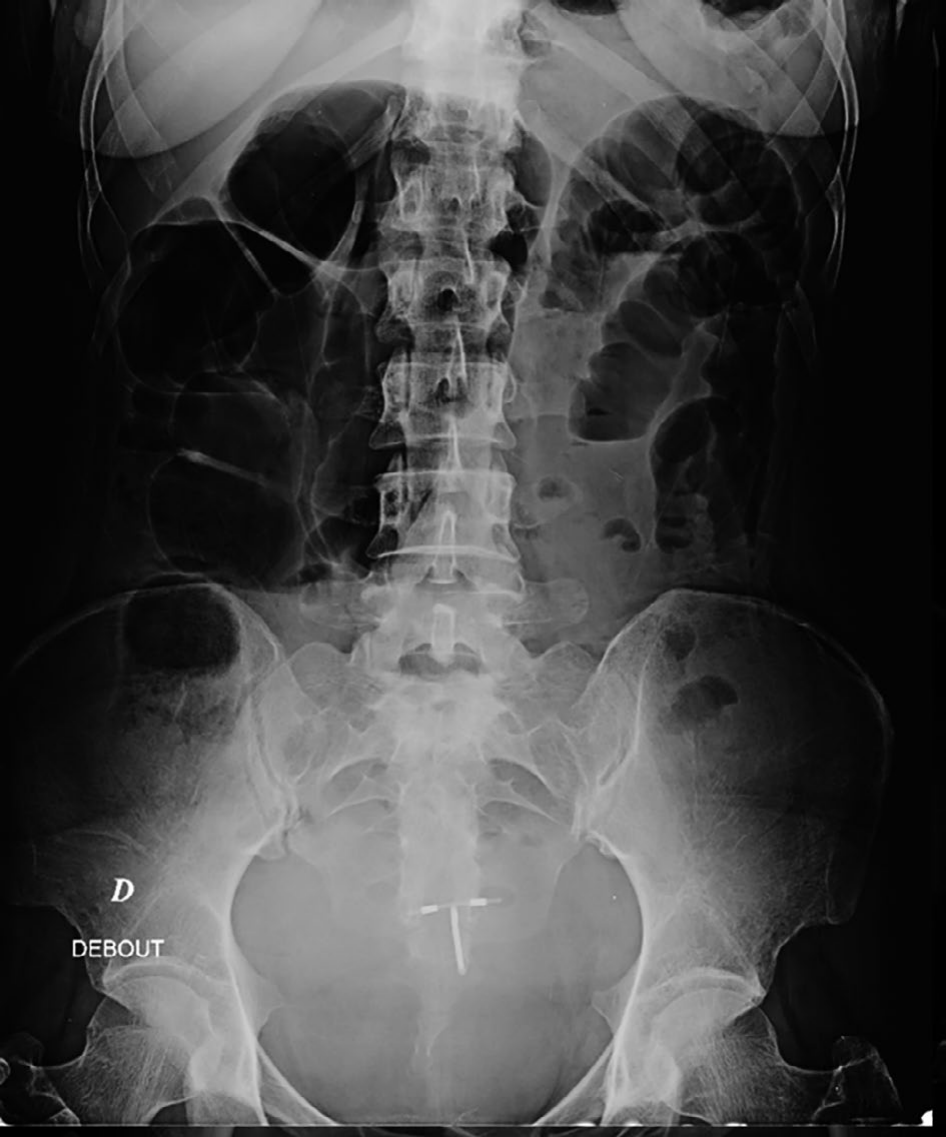
X‐ray image at presentation showing multiple air‐fluid levels

**FIGURE 2:**
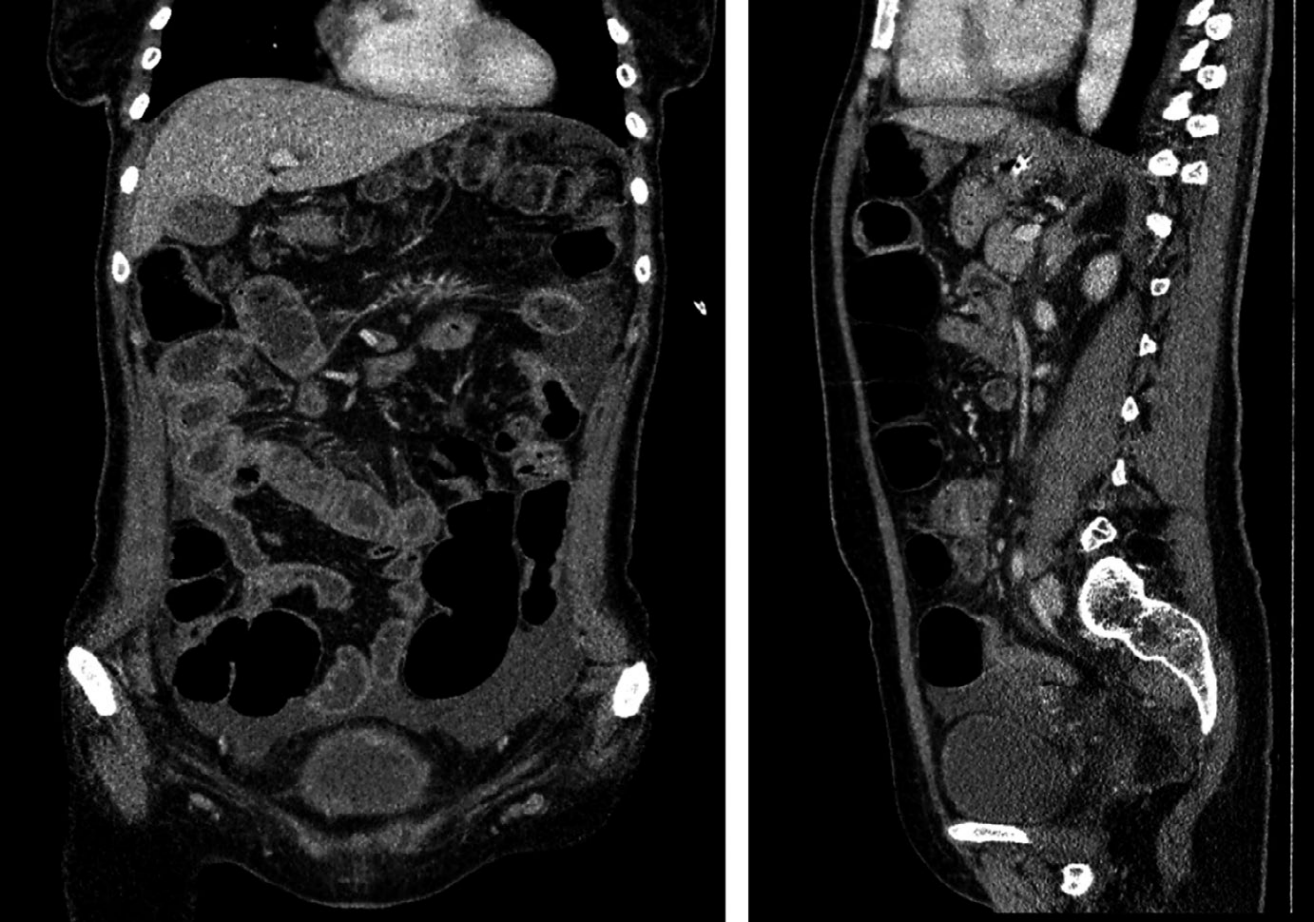
Abdominal CT scan views showing marked dilatation of small bowel loops and small intestinal wall thickening

A 37‐year‐old Caucasian female presented to the emergency room with abdominal pain, distension, and vomiting. Symptoms have appeared two months ago with reported diarrhea and weight loss. She already was treated symptomatically but no diagnosis was done and her condition went worse as she developed subacute bowl obstruction signs. She had no other previous significant personal history except polyarthralgia. No concomitant medication was taken. At presentation, physical examination revealed marked abdominal distension, diffuse tympanism, and tenderness without rebound tenderness. There was no fever, and vital signs were stable. Neurological and cutaneous examinations were normal. Abdominal X‐ray imaging showed multiple air‐fluid levels in small and large bowel (Figure 1). Investigations blood tests indicated normochromic anemia of 9 g/dL with reticulocytes count of 27 058/mm^3^, leucopenia of 3500/mm^3^, lymphopenia of 500/mm^3^, and normal platelets count. Blood chemistry tests showed: hypokaliemia of 2.6 mmol/L, sodium of 142 mmol/L, albumin of 2.8 g/dL, and normal lipase level. Thyroid function and hepatic tests were normal, and there was no inflammatory syndrome. Hemolysis markers were negative. Dipstick urinalysis showed normal results. Abdominal CT scan revealed small bowel dilated loops, segmental thickening of small intestinal wall without any mechanical obstacle (Figure 2), bilateral pleural, pelvic, abdominal effusion and not marked bilateral hydronephrosis. The patient was diagnosed with intestinal pseudo‐obstruction, and a nasogastric tube was placed. Antibiotics against bacterial growth, parenteral nutrition, and potassium supplementation were prescribed, as well as intravenous perfusion of erythromycin, leading to moderate improvement of symptoms. Colonoscopy was performed showing segmental thickening and edematous appearance of colonic mucosa with no specific findings at histopathology. Upper endoscopy was normal as well as histological examination of duodenal biopsies. Considering young age, female patient, and history of polyarthralgia, we investigated systemic diseases.

Immunological tests showed positive antinuclear antibody (ANA) at a titer of 1:400, anti‐dsDNA antibodies, anti‐SSB, and anti‐SSA. Direct Coombs' test was positive in the absence of hemolytic anemia.

Thus, she was diagnosed with SLE‐related IPO as she met 5 criteria of Systemic Lupus International Collaborating Clinics (SLICC) classification: leucopenia < 4000/mm^3^ / lymphopenia < 500/mm^3^, pleural effusion, positive Coombs' test without hemolytic anemia, ANA, and anti‐dsDNA antibody. Subsequently, we started corticosteroid therapy. The patient received 3 days methylprednisolone pulses (1000 mg per day) followed by oral prednisolone 1 mg/kg/day associated with Hydroxychloroquine (400 mg/day). Her condition had rapidly improved significantly with disappearance of abdominal symptoms, vomiting, and air‐fluid levels at X‐ray. The patient was discharged four days after starting corticosteroids. During follow‐up and after steroids tapering, the patient remained symptom‐free, continuing only Hydroxychloroquine by the last time she was reviewed 6 months after the onset.

## 3 DISCUSSION

Gastrointestinal involvement is not common among patients presenting SLE.^1^ It may include lupus mesenteric vasculitis, intestinal pseudo‐obstruction, protein‐losing enteropathy, pancreatitis, and hepatobiliary manifestations.^1^ Intestinal pseudo‐obstruction is a gut motility disorder and a rare condition when associated with SLE.

Pathogenesis of SLE‐related IPO remains unclear. It could be due to intestinal vasculitis affecting visceral smooth muscles or to autoantibodies targeting smooth muscle with immune complex deposition on the muscle and/or nerve, explaining gut dismotility disorder during SLE.^3^

The diagnosis of IPO is made based on clinical signs, air‐fluid levels, and bowel dilatation, with or without bowel wall thickening in imaging. Esophagogastroduodenoscopy, colonoscopy, and abdominal CT scan are performed to exclude mechanical obstruction.^4^ Intestinal manometry may be needed to exclude systemic sclerosis or Hirschprung's disease.^5^

Endoscopic mucosal biopsies are usually normal or show nonspecific findings because histopathological lesions of IPO are deeper located in the smooth muscle layer.^6^

Diagnosis might be challenging especially if IPO is the first manifestation of SLE.^3^ Indeed, misdiagnosis rate is very high, up to 78%.^2^

Ureterohydronephrosis, anti‐U1 RNP antibodies, peritonitis, and low C3 level were identified as being independent predictors of IPO in SLE.^7^

Thus, authors recommend regular abdominal X‐ray examinations in patients with these predictors. In our case, hydronephrosis was noticed at first presentation.

Supportive treatments of IPO include parenteral nutrition, prokinetics (neostigmine, erythromycin), and antibiotics against bacterial overgrowth.^8^ Furthermore, specific treatment of SLE‐related IPO is based on corticosteroid therapy ± associated with immunosuppressants (cyclophosphamide / azathioprine / tacrolimus).^3^, ^8^ In a review of the literature, a good response was obtained in 81% of patients who received corticosteroids as initial therapy.^3^ However, none of the patients who underwent surgical intervention achieved long‐term improvement.^3^, ^8^ In addition to ineffectiveness, unnecessary surgical intervention may cause additional medical issues. Nevertheless, urgent surgery must be indicated in acute complications of IPO such as intestinal perforation or ischemia.

It is important to timely initiate medical treatment; otherwise, the smooth muscle layer can progress to irreversible fibrosis.^9^

Regarding prognosis, IPO‐related SLE can lead to significant morbidity and mortality if not rapidly diagnosed and treated. According to another review of the literature,^8^ mortality rate was of 6.99% (10/143). Nephrotic syndrome, ureterohydronephrosis, and megacholedochus were found to be independent poor prognostic factors in SLE‐related IPO.^2^ In this report, although the patient had hydronephrosis, she presented favorable outcome.

In conclusion, we reported a case of IPO presenting as the initial manifestation of SLE with a spectacular response to steroids. Due to its rarity, diagnosis of SLE‐related IPO may be challenging especially if the underlying disease is not yet established. However, it is important to promptly recognize this rare condition since misdiagnosis can lead to unnecessary surgical intervention. Systemic diseases should be always considered when managing unexplained IPO, as medical treatment based on corticosteroids ± immunosuppressive therapy is very efficient.

## CONFLICT OF INTEREST

None declared.

## AUTHOR CONTRIBUTIONS

Dr Myriam Ayari: gathered the information, was involved in the definition of intellectual content, literature search and prepared the manuscript. Dr Abdelwaheb Nakhli: designed the concept, was involved in the definition of intellectual content, in the literature search and preparation of the manuscript. Dr Zeineb Teyeb: assisted in the preparation of the manuscript. Dr Imen Abdelaali: contributed to the critical review of the manuscript. Dr Syrine Bellakhal: extracted and interpreted imaging data from the medical records and contributed to the critical review of the manuscript. Dr Taieb Jomni: revised the manuscript and acted as guarantor for the research.

## ETHICS STATEMENT

Written informed consent was obtained from the patient for publication of this case report and any accompanying images.

## DATA AVAILABILITY STATEMENT

All data are available as part of the article and no additional source data are required.
